# First Human Model of *In Vitro Candida albicans* Persistence within Granuloma for the Reliable Study of Host-Fungi Interactions

**DOI:** 10.1371/journal.pone.0040185

**Published:** 2012-06-29

**Authors:** Nidia Alvarez-Rueda, Marjorie Albassier, Sophie Allain, Florence Deknuydt, Frédéric Altare, Patrice Le Pape

**Affiliations:** 1 Département de Parasitologie et de Mycologie Médicale, Université de Nantes, Nantes Atlantique Universités, EA1155– IICiMed, Faculté de Pharmacie de Nantes, France; 2 Laboratoire de Parasitologie-Mycologie, CHU de Nantes, Nantes, France; 3 CRCNA, Inserm U892, CNRS 6299, Université de Nantes, Nantes, France; Louisiana State University, United States of America

## Abstract

**Backgound:**

The balance between human innate immune system and *Candida albicans* virulence signaling mechanisms ultimately dictates the outcome of fungal invasiveness and its pathology. To better understand the pathophysiology and to identify fungal virulence-associated factors in the context of persistence in humans, complex models are indispensable. Although fungal virulence factors have been extensively studied *in vitro* and *in vivo* using different immune cell subsets and cell lines, it is unclear how *C. albicans* survives inside complex tissue granulomas.

**Methodology/Principal Finding:**

We developed an original model of *in vitro* human granuloma, reproducing the natural granulomatous response to *C. albicans*. Persistent granulomas were obtained when the ratio of phagocytes to fungi was high. This *in vitro* fungal granuloma mimics natural granulomas, with infected macrophages surrounded by helper and cytotoxic T lymphocytes. A small proportion of granulomas exhibited *C. albicans* hyphae. Histological and time-lapse analysis showed that *C. albicans* blastoconidia were located within the granulomas before hyphae formation. Using staining techniques, fungal load calculations, as well as confocal and scanning electron microscopy, we describe the kinetics of fungal granuloma formation. We provide the first direct evidence that *C. albicans* are not eliminated by immunocompetent cells inside *in vitro* human granulomas. In fact, after an initial candicidal period, the remaining yeast proliferate and persist under very complex immune responses.

**Conclusions/Significance:**

Using an original *in vitro* model of human fungal granuloma, we herein present the evidence that *C. albicans* persist and grow into immunocompetent granulomatous structures. These results will guide us towards a better understanding of fungal invasiveness and, henceforth, will also help in the development of better strategies for its control in human physiological conditions.

## Introduction

*Candida spp.* are polymorphic ascomycete fungi that grow either in a unicellular blastoconidia yeast form or as hyphae [1], [2]. They live as commensal organisms with normal mucosal or cutaneous microflora [3], [4]. In approximately half of the human population, *C. albicans* colonizes the gastrointestinal tract without any clinical symptoms [5]. However, prolonged chemotherapy, immunosuppressive treatment, or surgical intervention in intensive care units can turn this usually commensal yeast into a pathogen implicated in life-threatening acute invasive candidiasis [6]–[11].

In the context of chronic pathologies, the two main features of *Candida* infection are chronic disseminated candidiasis (CDC) also known as hepatosplenic candidiasis and chronic mucocutaneous candidiasis disease (CMCD). CMCD is characterized by persistent superficial infections of the skin, nails, oral and genital mucous membranes as well as impaired cell-mediated immunity against *C. albicans* [12]–[14]. CDC is present almost exclusively in patients with hematological malignancies such as acute and chronic lymphocytic leukemia. The risk of fungal complications increases with the duration of the leukemia, reflecting the natural history of the disease and the neutropenia linked to its treatment [15], [16]. The histological examinations of CMCD and CDC show evidence of granuloma structures composed of epithelioid histiocytes, multi-nucleated giant cells and lymphocytes with a chronic intensive inflammatory reaction around the yeast blastoconidia or hyphae [17]. Other studies have shown that granulomatous structures due to fungal infections remain rare, but are very aggressive and resistant to therapy [18]–[21]. The implication of inherent or acquired T-cell immunodeficiencies in these *C. albicans* infections has been recently demonstrated [14], [22], [23]. A better understanding of the molecular basis of fungal invasiveness within granulomas is essential for the development of better strategies for its control. However, because of the lack of clinically relevant models, the cellular organization and host-fungi interactions within the complex granulomas have not been well -described to date. To our knowledge there are no relevant models illustrating *in vitro* granulomas, leading to clinical extrapolation.

The invasiveness of *C. albicans* is very closely linked to the balance between the human innate immune system and the fungal virulence. This ultimately dictates the outcome of pathogenic dissemination [24], [25]. During the development of candidiasis, numerous types of immune cells including neutrophils, tissue macrophages, monocytes and dendritic cells interact with the fungus [26]–[28]. Most *in vitro* studies used a single cell type to infection with *C. albicans* [29], [30]. Other studies have described the infection of different cell subsets of human blood [31]–[34]. However, these cellular models do not reflect the multi-cellular organized structure of the granuloma. A first attempt to achieve this used of human Peripheral Blood Mononuclear Cells (PBMC) infected with heat-killed *C. albicans* [35]. Reconstituted epithelial tissues derived from human carcinomas are also used as “host-like” environments to study *C. albicans* virulence factors during adherence and invasion, or to follow protective immune cytokine production [36]. These models are often confronted with high concentrations of heat-killed *C. albicans* for infection. Consequently, limitations of current experimental methodologies do not permit the study of host-fungi interactions under pathophysiological conditions and over long periods.

It is highly desirable to develop relevant and complementary model systems in order to study the interactions between humans and fungi during local tissue inflammation and chronic disseminated infections. Likewise, the kinetics and nature of the cells that interact to form a structured microenvironment during *C. albicans* persistence are not fully understood.We hypothesize that *C. albicans* -structured granulomas represent a source of persistence and a way of propagating disease when mucosal barriers and blood immune systems become weakened. To develop a granulomatous model, we used freshly collected human PBMCs from healthy individuals. This allowed us to evaluate the relevance of an *in vitro* persistence model of *C. albicans* in human blood cell phagocytes in a cellular and physiological environment that mimics the *in vivo* situation. In this study, we induced a physiological granulomatous reaction in order to investigate the kinetics of cellular interactions during the very first stages of infection as well as during the late stages of *C. albicans* persistence.

## Results

### Development of a Cellular Aggregation Around Live *C. albicans*

**Figure 1:**
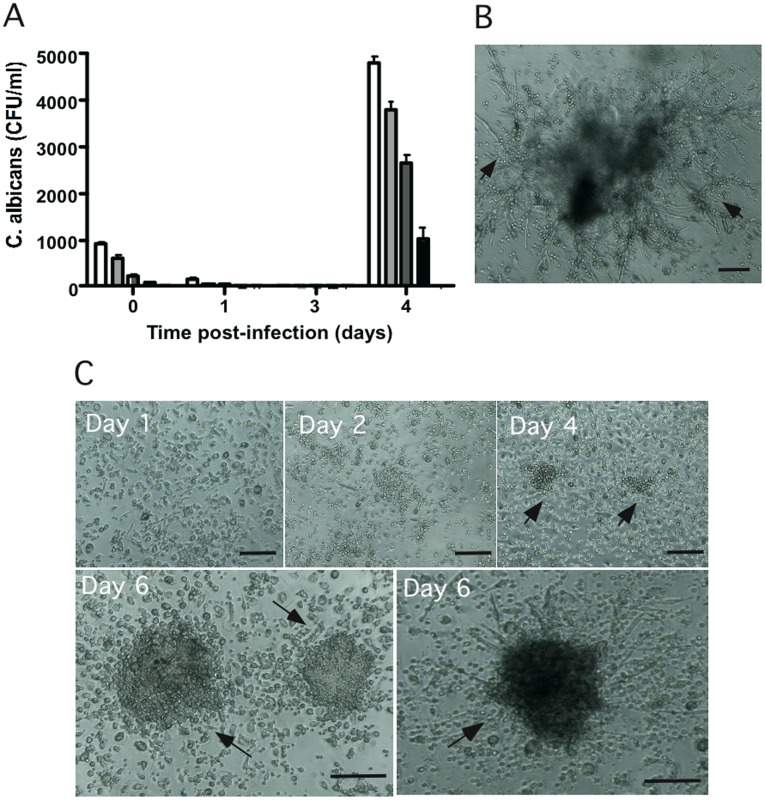
Recruitment of local human PBMCs around living *C. albicans.* (**A**)**:** Multiplicity of infection calculations (MOIs). 10^6^ cells/ml PBMC were infected with different concentrations of yeast cells and incubated at 37°C, 5% CO2. Cellular aggregation was followed every day under light microscopy. Uninfected PBMCs were used as a negative control of aggregation. The amount of surviving yeasts in granuloma structures at different time points was expressed as colony-forming units per ml (CFU/ml). The data are presented as the mean ± SD for three independent experiments, each in triplicate. MOIs tested were: 200∶1 (*white column*), 400∶1 (*light grey column*), 800∶1 (*grey column*) and 2000∶1 (*black column*). (**B**)**:** Light-microscopic observation of a representative granuloma six days after *C. albicans* infection (MOI of 400∶1). Large hyphae envading from granuloma (arrows). Bar represents 50 µm. (**C**)**:** Light microscopy observation of time course evolution of the granulomatous reaction after infection with *C. albicans* (MOI of 2000∶1). Bars represent 50 µm.

The first step in the development of an *in vitro* human model of *C. albicans* granuloma was to induce recruitment of peripheral blood mononuclear cells around live *C. albicans* blastoconidia. We first determined the optimal multiplicity of infection (MOI) of *C. albicans* for use in subsequent experiments. The monocyte:yeast MOIs ratios studied were 200∶1, 400∶1, 800∶1 and 2000∶1. These experiments showed that phagocytes displayed high candidicidal activity during the first 3 days after *C. albicans* (CAAL93) infection (Figure 1A). For all MOIs, the surviving yeasts were then responsible for a significant and rapid increase in the fungal load from the fourth day post-infection. However, the MOIs 200∶1, 400∶1 and 800∶1 caused premature destruction of granulomatous structures due to an uncontrolled increase in the fungal load as numerous *C. albicans* hyphae invaded the plate wells (Figure 1B, arrows). By contrast, highest MOI of 2000∶1, *C. albicans* proliferation also induced a significant number of colony-forming units 4 days after infection (1033±284 CFU/ml). Interestingly, at this MOI a cellular aggregation resembling to granuloma structures were observed under light microscopy. There was also a better capacity for limiting the spread of hyphae with the highest MOI. These host cells and yeast aggregates remained under control until the 6th day after infection (Figure 1C). At this point, the presence of a limited proportion of hyphae colonized structures (Figure 1C, right image) explained in part the higher fungal load (4000±179 CFU/ml). The MOI of 2000∶1 was chosen for use in subsequent experiments.

### Characterization of *C. albicans* Elicited Granuloma

**Figure 2:**
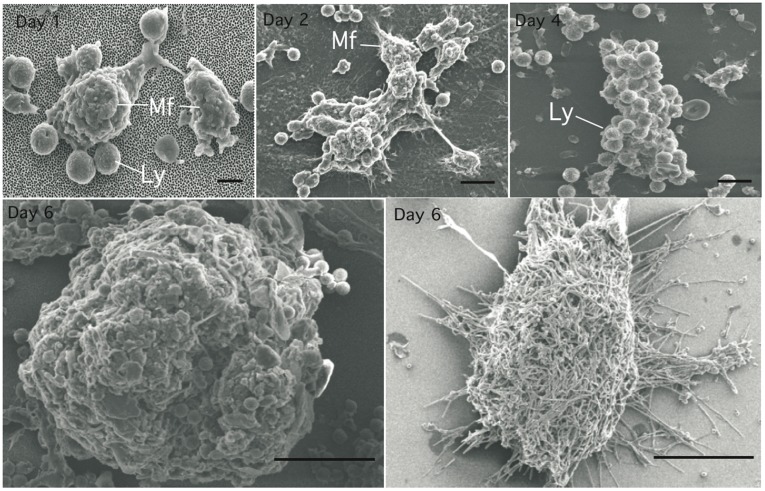
Fungal granuloma progression by scanning electron microscopy. Kinetics of granuloma formation after infection with *C. albicans* (MOI of 2000∶1). The granuloma structures were collected at different time points from co-culture plates, and fixed in 2.5% glutaraldehyde, O.1 M sucrose in cacodylate buffer for 1 h. After dehydratation through a gradient ethanol series and alcohol-freon substitution, specimens were coated with 100 Å of gold-palladium mix in an ion sputter and photographed under a scanning electron microscope. Bars represent 5 µm (Days 1 post-infection), 10 µm (days 2 and 4) and 50 µm (day 6). Activated macrophages *(Mf)*, lymphocytes *(Ly).*

The progression in size of these cellular aggregates was followed using scanning electron microscopy (SEM), as shown in Figure 2. Between days 1 and 3 after infection, monocyte-like cells appeared to begin to assemble, then lymphocytes rapidly flattened themselves around the monocyte-like cells. Progressively, between days 4 and 6 after infection, multi-layers made up of macrophages and lymphocytes became more complex and compact, exhibiting the characteristics of a typical granuloma. Interestingly, a matrix of extra-cellular polymeric substance was observed under SEM, in particular when the granuloma structures were quite mature or the blastoconidia had filamented (Figure 2, day 6 right image).

**Figure 3:**
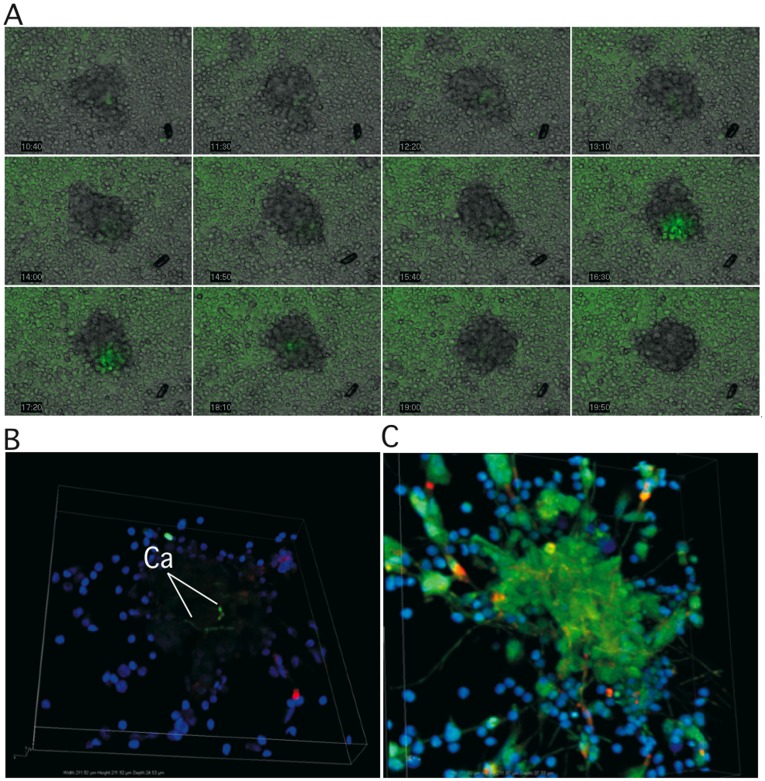
Distribution of *C. albicans* cells in the granuloma. (**A**)**:** For co-culture analysis by video-imaging, PBMCs were infected with *C. albicans* cells containing the pACT1-GFP fusion protein at a MOI of 200∶1. The cells were illuminated at day 0 and every 10 min over 72 h of incubation with a 300 W xenon lamp fitted with 488 nm excitation filter. Emission at 515 nm was used for analysis of *C. albicans* fluorescence with a Leica DMI6000B camera cool Snap HQ2 and processed with Metamorph imaging software version 7.7.4.0. (**B and C**)**:** For confocal microscopy imaging granulomas were generated by infecting PBMCs at an MOI of 2000∶1. Cells were stained with rhodamine-phalloidin and Hoechst six days after infection. Confocal images showed GFP- tagged *C. abicans* inside granulomas (**B**). Large green-fluorescent hyphae of *C. abicans* emerging from granulomas (**C**)**.** Image scan was 1024 pixels × 1024 pixels. Image size was 1212 µm.

The kinetics of granuloma formation were also studied using time-lapse fluorescence microscopy and confocal microscopy. These microscopy techniques were also used to localize GFP-tagged *C. albicans* in the structures. Figure 3A shows a representative time-lapse sequence photographed by combined confocal and bright-field imaging over 72 hours after infection. The sequence showed that PBMCs began to collect around GFP-tagged *C. albicans* cells, with the progressive formation of a granulomatous structure. Fluorescence levels were low at this stage, suggesting that the yeasts were distributed within the granulomas. Interestingly, the fusion of two still-forming granulomas was generally observed in all the donors (Video S1).

To more readily observe the yeasts, we generated granulomas by infecting PBMCs at an MOI of 2000∶1. We examined their distribution 6 days after infection using confocal microscopy after staining with rhodamine-phalloidin and Hoechst. *C. albicans* cells were mainly observed inside granulomas, where they appeared to be associated with the core (Figure 3B and Video S2). They were also observed in the two morphological structures, such as blastoconidia or as hyphae. In some cases, large hyphae emerged from the granulomas. PBMCs appeared isolated in areas largely invaded by *C. albicans* hyphae (Figure 3C and Video S3).

**Figure 4:**
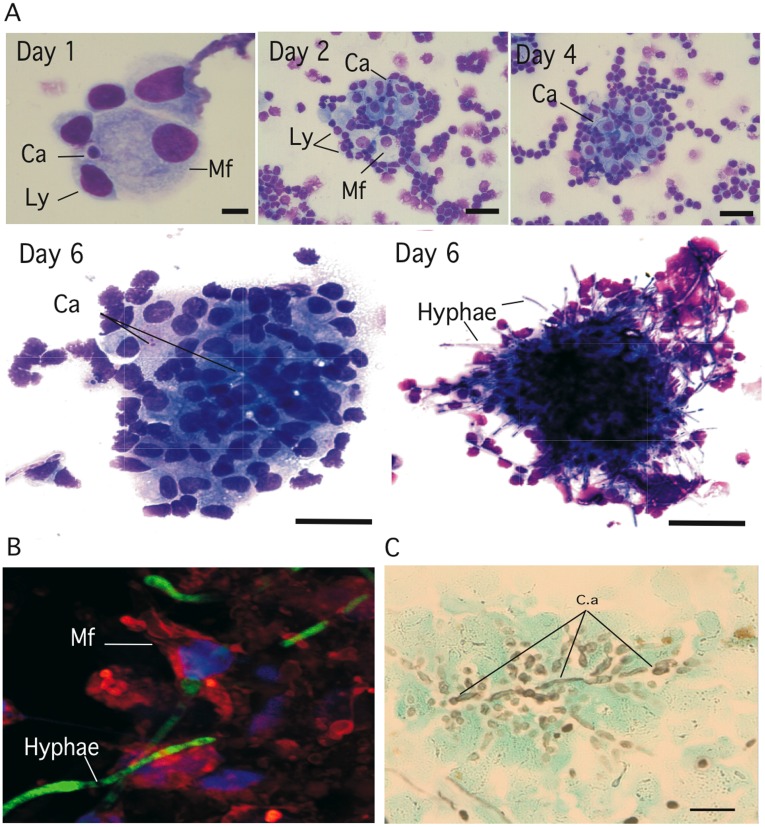
Histological analysis of human cell subsets surrounding fungal granulomas. Granuloma structures were collected at different time points of incubation**,** plated on glass slides with a cytospin and stained. (**A**)**:** May-Grünwald-Giemsa (MGG, Sigma) staining of granulomas at different time points. Bars represent 5 µm (Day 1), 15****µm (day 2 and 4) and 50 µm (day 6). (**B**)**:** Confocal images showing the interaction between mature macrophages and green-fluorescent *C. abicans* hyphae inside granulomas. (**C**)**:** Grocott-Gomori methenamine silver nitrate staining of a granuloma six days post-infection (bar represent 50 µm). Mature macrophages (*Mf*), lymphocytes *(Ly), C. albicans (C.a).*

*C. albicans* polymorphism and cell populations of human PBMC recruited after infection were characterized after plating the granuloma structures by cytospin and staining with May-Grünwald-Giemsa (MGG). Representative histological observations are shown in Figure 4. Four-day-old granuloma structures were mainly characterized by the presence of yeast-infected macrophages and/or activated macrophages (Mf) with large vesicles: few cells appeared multi-nucleated. Lymphoid cells resided mainly at the periphery of infected macrophages or infiltrate the outer layer of the induced granuloma. After 6 days of culture, lymphoid cells (Ly) and activated macrophages (Mf) displayed a multilayer organization and the classical appearance of a tightened granuloma. At this stage, cellular infiltration was extremely apparent and was characterized by very large zones of migrating cells, surrounding the granuloma structures (Figure 4A). Confocal microscopy analysis confirmed that compacted aggregates of mature macrophages (Mf) interacted with yeasts and hyphae (Figure 4B, Video S4 and S5). Mature macrophages were characterized by their increased cytoplasmic size and by their ruffled cell membranes that link the adjacent cells. When stained with Grocott-Gomori methenamine silver nitrate, these granuloma structures revealed fungal burdens with the presence of hyphae (Figure 4C).

### Capacity of *C. albicans* Clinical Isolates to Induce an *in vitro* Granulomatous Response

**Figure 5:**
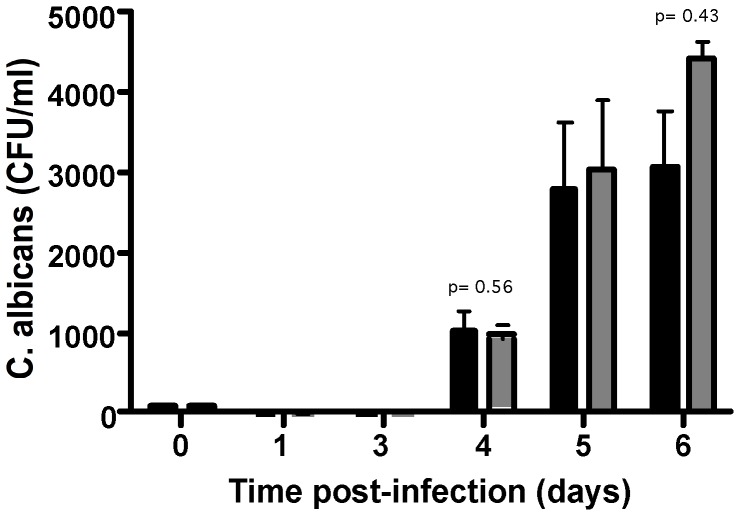
Capability to *C. albicans* clinical isolates to generate granulomas. Granuloma formation by different *C. albicans* clinical isolates. Human PBMCs were infected with CAAL93 (*black columns*) and DSY735 (*grey columns*) clinical isolates. Granuloma formation was followed by light microscopy. The fungal load was expressed as CFU/ml at different incubation times. The data are presented as the mean ± SD for three independent experiments, each in triplicate.

To determine whether the induction of PBMC aggregation is isolate-specific, we repeated the above experiments at a MOI of 2000∶1 by comparing CAAL93 and DSY735 clinical isolates. These *C. albicans* strains have been previously characterized for their comparable *in vivo* virulence [37]. Interestingly, we found that PBMC from healthy donors were not able to completely eliminate *C. albicans* infection. As shown in Figure 5, after initial candidicidal activity, both CAAL93 and DSY735 *C. albicans* strains induced similarly high numbers of colony-forming units even on day 4 after infection. However, the number of colony-forming units was higher on day 6 after infection. Light-microscopy observation at this time point showed the presence of some aggregates with fungal hyphae corroborating the high CFU levels (data not shown).

### *In vitro* Fungal Granulomatous Response is a Phenomenon Conserved in Healthy Donors

**Figure 6:**
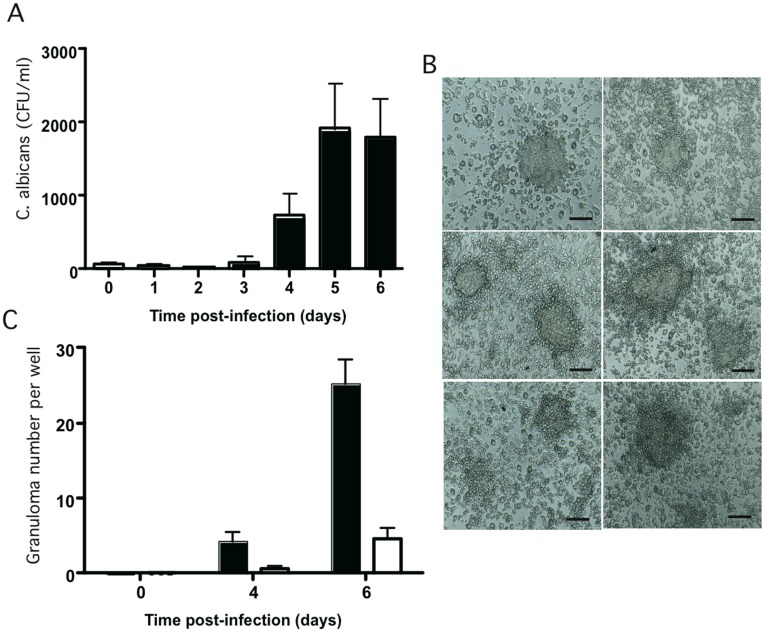
Fungal granuloma-response variability between donors. (**A**)**:** The fungal granuloma-response variability between healthy individuals was analyzed by infecting the PBMCs from 23 donors with *C. albicans* (MOI of 2000∶1). The granuloma-response was followed by calculating the colony-forming units (CFU/ml) at different time points and by light microscopy observations. The data are presented as the mean ± SD for 23 independent experiments. (**B**)**:** Light-microscopic observation of representative granulomas from donors after six days of infection with *C. albicans*. Bar represents 50 µm. (**C**)**:** Number of specific granulomas per culture-well at different times of infection with *C. albicans*. Intact granulomas *(black columns)*, granulomas with *C. albicans* hyphae *(white columns).*

Human PBMCs in the above experiments were specifically capable of responding to two *C. albicans* strains. It is possible, however, that where complex mixtures of immunocytes interact, the variability between individuals may influence the progression of the granulomatous response. Thus, we incubated human whole PBMCs from 23 healthy donors with *C. albicans* (CAAL93) and followed the granuloma formation over 6 and 7 days. As shown in Figure 6A, the kinetics of the fungal load after infection were comparable among the donors (*p* = 0.220 between days 5 and 6). After initial candidicidal activity of phagocytes at the earlier stages of cell aggregation (between day 1 and 3), an increase in CFU number between days 4 and 6 reflected *C. albicans* multiplication and its persistence in granuloma structures. Multi-layer cellular accumulations with differentiated cellular structures were formed in every healthy donor tested. This granulomatous response was also comparable across donors, both in terms of cell attraction around *C. albicans* yeasts and qualitative cellular recruitment (Figure 6B).

Light-microscopy observation of all the donors at different incubation time points confirmed that the polarized growth of *C. albicans* occasionally rupture some of the granuloma structures by producing hyphae. However, six days after infection, this escape phenomenon affected a small proportion of granuloma structures (1.7±0.3/well) compared to intact granuloma structures (8±2/well) (Figure 6C).

### Characterization of Lymphocyte Subsets into Granulomatous Structures

**Figure 7:**
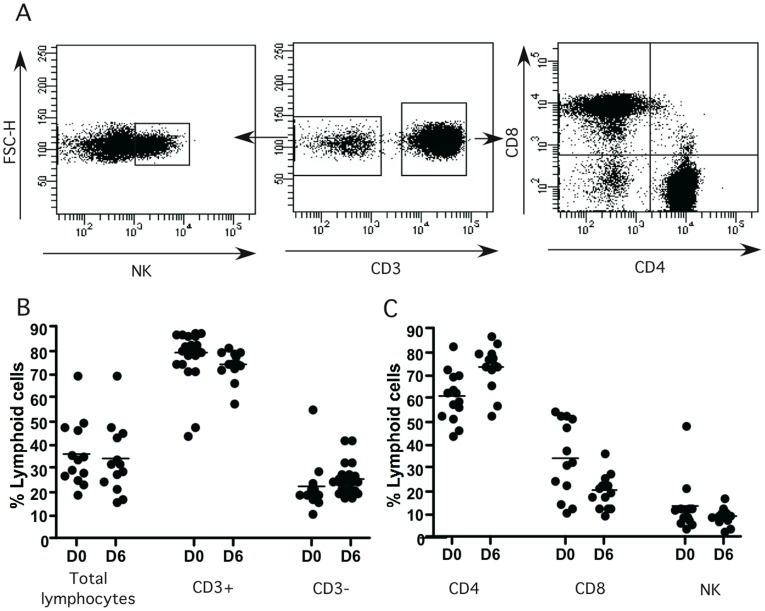
Recruitment of cell subsets in fungal granuloma. The granuloma structures were collected at different times points from co-culture plates under light microscopy, and stained with a cocktail of fluorescent-conjugated antibodies specific to CD3, CD4, CD8, and NKp46. (**A**)**:** The cells were analyzed by flow cytometry after gating on CD3 lymphocytes: the CD3+ population was separated into conventional CD4+ and CD8+ T cells. Natural killer cells were gated from the CD3- population. (**B**)**:** Total lymphocytes, CD3+ and CD3- subsets percentages into granulomas for each donor on day 0 (D0) and day 6 (D6) (n = 13). (**C**)**:** CD4+, CD8+ and NK lymphocyte cells into granulomas (n = 13 donors). All data were acquired using a FACS Canto II instrument (BD Biosciences) and analyzed with FlowJo software version 9.4.10 (Tree Star Inc) and DIVA software version 6.2 (BD).

We then used flow cytometry to analyze the lymphocyte subsets, which migrated around the *C. albicans* infected macrophages. The granuloma structures from 13 healthy donors were isolated from co-culture plates six days after infection with *C. albicans* (CAAL93). A representative lymphocyte flow cytometry analysis from the donors is shown in Figure 7A. The total number of lymphocytes, T cells (CD3+), helper T cells (CD3+CD4+), cytotoxic T cells (CD3+CD8+) and NK cells (Nkp46+CD3-) were analyzed. The means and standard deviations of each lymphocyte subset on day 0 before *C. albicans* infection and 6 days after are presented in Figure 7B and 7C. The healthy donors used in this study showed no significant difference in all populations on day 0 before the infection with *C. albicans*. Analysis of lymphocytes six days after infection showed that a total of 33.2%±4.4 of the cells surrounding granulomas were lymphocytes. Among these, 74.5%±1.8 were T lymphocytes. Among these, 74.7%±2.7 were T helper cells and 20.3%±2.1 were cytotoxic T cells. Interestingly, we observed a significant increase of the percentage of the granuloma infiltrated CD4+ T lymphocytes compared to the uninfected control on day 0 (74.7%±2.7 vs. 61.1%±2.8, *p* = 0.0038). Consequently, there was a significant reduction of the percentage of infiltrated CD8+ T lymphocytes (20.3%±2.1 vs. 34.6%±4.5, *p* = 0.0087). The NK cell subset was 9.5%±1 within the *C. albicans-* generated granuloma without significant differences compared to control on day 0. The uninfected controls six days after incubation did not show significant differences on T cell populations compared to the control on day 0 (data not shown).

### Impact of Polymorphonuclear Neutrophilic Granulocytes (PMNs) on Granuloma Formation

**Figure 8:**
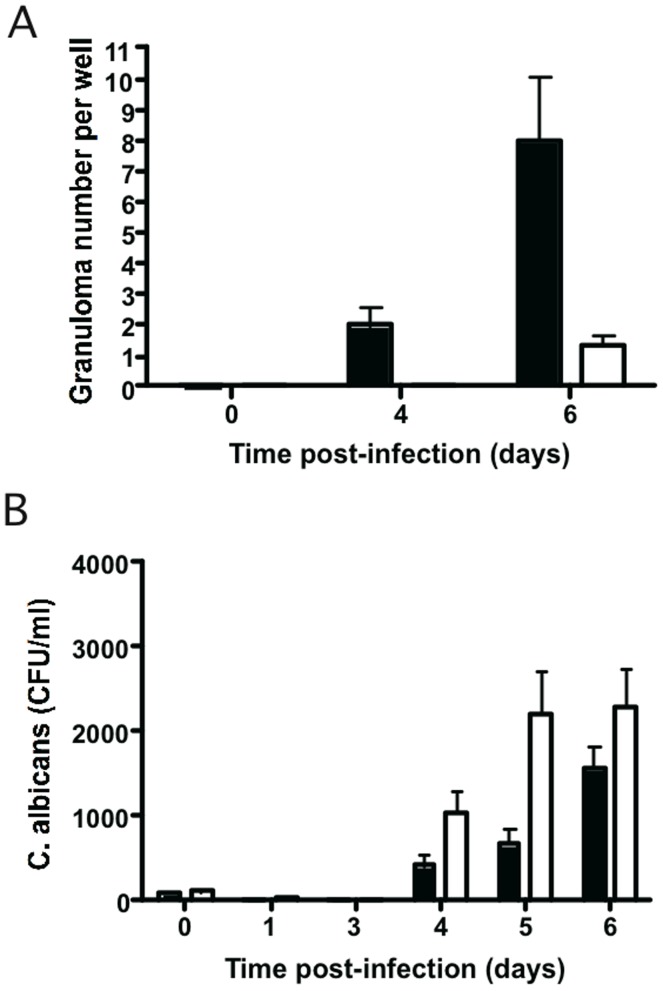
Effect of PMNs on granuloma formation. PBMCs alone or PBMCs with PMNs from healthy individuals were used to induce *in vitro* granulomas after *C. albicans* (CAAL93) infection. Host cells were infected with yeasts at an MOI of 2000∶1 and incubated at 37°C, 5% CO2. Cellular aggregation was followed every day under light microscopy. Uninfected PBMCs and PMNs were used as a negative control of aggregation. (**A**)**:** The number of granulomas per well was followed at different time points. Intact granulomas (*black columns*), granulomas with *C. albicans* hyphae (*white columns*). (**B**)**:** The amount of surviving yeasts in granuloma structures at different time points was expressed as colony-forming units per ml (CFU/ml). PBMCs with PMNs co-cultures (*black columns*), PBMCs co-cultures (*white columns*). The data are presented as the mean ± SD, each experiment in triplicate (n = 5).

PBMCs alone or PBMCs with PMNs from healthy individuals were used to induce *in vitro* granulomas after *C. albicans* (CAAL93) infection. Granuloma structures were compared at different time points in terms of fungal load and granuloma numbers. When PMNs were present among the PBMCs on day 0, a significant reduction in the number of granulomas (Figure 8A) and fungal loads (Figure 8B) was observed six days after infection compared to PBMCs without PMN conditions. Despite this reduction, yeasts persist however within granulomas and in some cases they differentiated into hyphae. Confocal microscopy analyses showed the same cellular organization of granulomas obtained after infection of PBMC (Video S6).

## Discussion

Understanding the molecular mechanisms underlying the interaction between fungi and host cells is essential in order to explain the saprophytic/pathologic balance of fungi species. Considering the above, it is highly desirable to develop relevant and complementary model systems to study host-fungi interactions.

The key finding of our study is that after candidicidal activity of human phagocytes, *C. albicans* is able to persist during the first days after infection of human peripheral blood cells *in vitro*. Yeasts then proliferate into granulomas providing the evidence of its ability to persist in healthy individuals, even in the presence of immunocompetent cells. Our observations are consistent with a significant role of PMNs as key host effectors on *C. albicans* granuloma formation early after infection, reducing the amount of *in vitro* granulomas and the invasion by *C. albicans* hyphae. In addition, the average lifetime of mature circulating PMNs being around 12 hours could suggest that *C. albicans* are rapidly phagocytosed and killed. However, as reported previously, the small amount of activating lymphokines that are produced under *in vitro* inflammatory conditions could lead to the prolonged lifespan of PMNs [38]–[40]. Further work is needed to define the effect of phagocytes on granuloma progression and/or resolution. These findings could lead us to better comprehend what happens during chronic candidiasis,

To the best of our knowledge, this is the first direct evidence that a fungal pathogen induces granuloma structures *in vitro*. Through a combination of histological staining, scanning microscopy, time-lapse microscopy and flow cytometry, we have provided diverse evidence supporting these findings. We found, however, that granulomas successfully form only when very low concentrations of blastoconidia are used to infect human phagocytes. The granuloma-response resembled chronic human pathology, where pathogens persist in tissue at low levels. This contrasts with previous models using cell lines or human peripheral blood cells [31]–[34], [41], [42], which utilized high concentrations of living or dead *C. albicans*.

Under our methodological conditions, a very small proportion of *C. albicans* eventually escape by forming hyphae. Our study suggests that the rapid adaptation of a small proportion of *C. albicans* population through yeast- hyphae switching could be an evolutionary strategy to facilitate movement from granulomas to blood vessels. However, previous observations showed that the non-filamentous *C. glabrata* is also able to survive and persist in immunocompetent mouse organs [43]–[45]. The secondary dissemination of this *Candida* species could depend less on its phenotypic switching capability.

*C. albicans* virulence mechanisms and host cell interactions have been well-documented with the use of different cell lines and animal models [29], [30], [36], [46], [47]. However, the demonstration that *C. albicans* can survive and persist in human immunocompetent tissue structures is poorly documented in that literature. Some studies described the degradation of *C. albicans* in murine hepatic macrophages [41]. Other *in vitro* studies used the infection of fractionated blood phagocytes by *C. albicans* in order to test yeast transcriptional variations as a function of the tested phagocytes [31]–[34], [41], [42]. Some studies have used an *in vitro* granuloma model with human PBMCs [48], [49] to investigate granulomatous hypersensibility caused by antigens from the dimorphic fungus *Paracoccidioides brasiliensis*. Heinemann at al. [35] proposed an *in vitro* model using heat-killed *C. albicans*; dead yeast were chosen because the cell wall glucans resist degradation. They described the role of peripheral blood monocyte proliferation after infection and the formation of irregular cellular aggregates; however, there were no granulomas when lymphocytes were added to the cultures. In addition, no investigation of the interaction between live *C. albicans* and the host cells under granuloma contitions was ever reported. In contrast to these previous studies, we developed a model in which the number of phagocyted *C. albicans* by monocytes was controlled, delaying the invasion and the rapid spread of filamented yeasts from phagocytes, thus enabling their interaction and subsequent granuloma formation. Our results also confirm earlier observations in CDC patients that *Candida spp.* burden can be very low, but yeasts can then be successfully re-isolated many days after infection [43]. This persistence could simulate the physiological phenomenon of dynamic latency in patients with CDC.

Granuloma formation is an organized, cell-mediated immune response mainly dependent on monocytes and T cells. It is well-recognized as a central feature of the pathogenesis in humans. Well-known infectious granulomatous diseases include tuberculosis, leprosy, schistosomiasis, sarcoidosis, and leishmaniasis [50]–[55]. Granulomas were characterized by the presence of yeast-infected macrophages and mature macrophages. Lastly, the migration of many cytotoxic and helper T cells was observed. Interestingly, NK cells were also recruited to contribute to the formation of granulomas. These morphological and cellular characteristics are similar to those of *in vivo* granulomas [53]. This *in vitro* human fungal granuloma model enables the analysis of the human granulomatous response against other fungal pathogens [48], [49]. By taking into account the representative immune cells, cell maturation studies can be suitably adapted and cell trafficking can be followed. These studies may be carried out to see if particular cells can migrate to the granuloma and subsequently leave these structures. Further work is needed to define the balance between pro- and anti-inflammatory cytokines that provides efficient protection from invasive fungal infection.

These data have important implications both for the clinical relevance of fungal granulomas and for the understanding of other pathophysiological processes involved in fungal persistence in humans. The clinical relevance of these observations is consistent with other recent European studies and clinical case reports, which have documented the presence of fungi in granulomatous structures [18]–[21]. It is clear from these studies that the clinical prognosis is often compromised when patients develop fungal granulomas, such as *Candida spp., Cryptococcus spp*., and *Aspergillus spp.,* leading us to suggest that granulomas could be a source of fungal persistence and antifungal resistance [18]–[20], [56]–[59].

The pathogenesis of chronic disseminated candidiasis is not well understood in humans. It seems that the invasion of *Candida* species from the gastrointestinal tract into the bloodstream, as a consequence of prolonged neutropenia and a breach in mucosal integrity, induces prominent *Candida* granulomas in the liver. Our results suggest that under these conditions *C. albicans* could survive at low levels–representing a source of persistence and chronicity–and then proliferate, leading to invasive disease propagation. That brings us to the question of how *in vivo* fungal granulomas function in order to limit infection, or how they can become a mechanism of resistance to anti-fungal therapy. Our results describe the creation of an organized human cellular response around yeasts, consistent with the granulomatous structures observed in patients. To our knowledge, the histological description of lesions from patients with localized or disseminated fungal infections is a narrow field of study in the medical community. Indeed, there is limited information about *in vivo* fungal granuloma in the literature.

From the data presented above, it is evident that our concept of *C. albicans* virulence mechanisms and host immune responses is closely related to granulomatous cellular organization. The use of different microscopy techniques has allowed for the first descriptive image of the host cell components and *C. albicans* localization in granulomas. Finally, it should be stressed that this first evidence that *C. albicans* can be organized into physiological tissue-structured communities *in vitro* could lead to a better understanding of the molecular basis of the persistence, invasiveness and aggressiveness of the fungus and, consequently, to the development of better strategies for its control.

## Materials and Methods

### Cells and Plasmids

The *C. albicans* CAAL93 clinical isolate came from the Mycobank of the Parasitology and Medical Mycology Department, Nantes, France. The *C. albicans* DSY735 clinical isolate was kindly provided by Pr. Dominique Sanglard from the Institute of Microbiology, UNIL, University of Laussane [60]. Virulence of *C. albicans* strains have been previously characterized. CAAL93 and DSY735 were highly virulent in an *in vivo* mouse model of disseminated candidiasis. pGFP and pACT1 plasmids were kindly provided by Pr. Alistair Brown from the School of Medical Sciences, University of Aberdeen, Institute of Medical Sciences, Aberdeen. *Escherichia coli* KL1-blue (Stratagen) competent cells were used for the transformation and propagation of recombinant pGFP and pACT1 plasmids. The *C. albicans* CAI4 (ura-) strain and the CIp-TDH3p-GFP in CAI4 (ura+) strain, were kindly provided by Pr. Christophe D’Enfert, from the Fungal Biology and Pathogenicity Unit of the Institut Pasteur, Paris, France.

### Preparation of *Candida albicans* Blastoconidia Suspensions

CAAL93 strain was streaked onto YPD agar (1% yeast extract, 2% peptone, 2% dextrose, 2% agar) and incubated overnight at 30°C. One colony was transferred to 10 ml of YPD liquid media and the cells were incubated overnight at 30°C in a shaking incubator. After washing twice in phosphate-buffered saline, the cells were suspended in RPMI 1640 (Sigma) with 8% heat-inactivated pooled human serum at a concentration of 10^6^ cells/ml. For co-culture experiments, *C. albicans* cells were counted in order to set up different Multiplicities Of Infection (MOI).

### Human Peripheral Blood Mononuclear Cells and *Candida albicans* Co-culture

Peripheral blood samples were obtained from healthy volunteers at the Etablissement Français du Sang, Pays de la Loire (Nantes, France). Peripheral blood mononuclear cells (PBMC) were isolated by gradient density sedimentation, using LMS 1077 (PAA Laboratories, Austria) lymphocyte separation medium as described by [61]. In some experiments polymorphonuclear neutrophilic granulocytes (PMNs) from donors were also isolated by gradient density sedimentation and added to PBMC, in order to test the effect of PMN on granuloma formation. All the analyses presented were performed according to the principle expressed in the Helsinki Declaration. Co-culture protocols were based on previously described *Mycobacterium tuberculosis* granuloma experiments by Altare et al., [62]. In brief, PBMCs were adjusted to a final concentration of 10^6^ cells/ml per each well. Then, yeast cells were added to the blood cells at different MOIs and incubated at 37°C, 5% CO2. Cellular aggregation was followed daily using light microscopy. Uninfected PBMCs were used as a negative control. At various time points of incubation, granuloma structures were processed for microscopic observation, as well as for determining the amount of surviving yeast in the granuloma structures and for flow cytometric analysis.

### Colony-forming Unit Assay

Candidicidal activity of human PBMC was measured by counting the living yeasts with a colony-counting technique (colony-forming unit, CFU). After co-culture at different time points, the cells were washed three times with phosphate-buffered saline. Phagocytes in the wells were lysed with Trypsine-EDTA to release the internalized yeasts. Serial dilutions were determined according to the original yeast number in the culture. Suspensions were spread on YPD plates and *C. albicans* colonies were counted after 24 h of incubation at 30°C. The CFU/ml data obtained corresponded to the fungal load of both yeasts within the granulomas and after hyphae formation.

### Scanning Electron Microscopy (SEM)

The granuloma structures from co-culture plates were collected at different time points and fixed in 2.5% glutaraldehyde, 0.1 M sucrose in cacodylate buffer for 1 h. The cells were washed twice in the same buffer and then fixed with 1% Osmium in cacodylate buffer for 1 h. After dehydration through a gradient ethanol series and alcohol-freon substitution, the specimens were coated with 100 Å of gold-palladium mix in an ion sputter (JEOL JFC 1100). The specimens were observed and photographed under a scanning electron microscope (JEOL 6400F).

### Video Imaging

For co-culture analyzes by video imaging, PBMCs were infected with CAI4 *C. albicans* cells containing the pACT1-GFP fusion protein. Ten µg of pACT1-GFP and pGFP fluorescence negative control plasmids were linearised by digestion with BglII and used to transform *C. albicans* by electroporation. Single copy integrants at the RPS10 locus were selected as previously described by Barelle et al., [63]. Single colony transformants from Minimal medium (SD) containing 2% glucose were inoculated into 1 ml of synthetic complete-medium containing CAS amino-acids in order to induce ACT1-GFP protein expression. The cells were incubated for 2 h at 30°C to reach maximum fluorescence. The cells were collected by centrifugation at 3000 *g* for 10 min, analyzed to find the expression level under fluorescence microscopy (Leica Microsystems, Nanterre, France), and then used to infect human PBMCs at a MOI of 200∶1 as described above. This MOI was chosen in order to increase the probability of finding and recording the formation of granulomas. The cells were illuminated at day 0 and every 10 min over 72 h of incubation with a 300 W xenon lamp equipped with a 488 nm excitation filter. Emission at 515 nm was used for analysis of *C. albicans* fluorescence with a Leica DMI6000B camera cool Snap HQ2 (Roper, Tucson, AZ) and processed with Metamorph imaging software version 7.7.4.0 (Universal Imaging, Downington, PA).

### Confocal Microscopy Imaging

GFP-tagged *C. albicans* induced granulomas in Lab-Teck™ slides were washed twice in phosphate buffered saline (PBS) and fixed with 4% paraformaldehyde for 30 min. After cell permeabilization with 100% acetone, nonspecific binding sites were blocked with 1% BSA in PBS for 30 min. Rhodamine-conjugated phalloidin (Wako; Osaka, Japan) was added at a 1∶600 dilution to stain actin filaments for 30 min at room temperature. Nuclear DNA was stained with Hoechst in PBS for 1 min. Slides were air-dried and mounted with Vectashield media. Fluorescence-stained sections were examined under a Nikon A1 RSI microscope with 20× magnification at constant Z-steps of 1 µm. Laser confocal system comprises a 65 mW Multi-Ar laser (405, 488 and 551-nm excitations are possible). 3D Images were processed with NIS elements version 3.21 (Nikon Instruments Inc.) and Volocity 3D Image Analysis Software version 6.01 (PerkinElmer).

### Histological Analyses

Granuloma structures were washed twice in phosphate buffered saline containing 2% FBS, collected at different time points of incubation and plated on glass slides with a cytospin (Cytobuckets S, Jouan). Cells were submitted to May-Grünwald-Giemsa (MGG, Sigma) and Grocott-Gomori methenamine silver nitrate. The stained slides were observed using light microscopy (Zeiss Scope A1) and phagocytosis and fungal-phagocyte interactions around the granuloma structures were identified.

### Flow Cytometry

The granuloma structures from co-culture plates were washed twice in phosphate buffered saline containing 2% FBS and collected at different time points under light microscopy. The cells were suspended in the same buffer to a final volume of 200 µl and stained with a cocktail of fluorescent-conjugated antibodies. The antibodies were specific to CD3-Pecy7 (clone UCHT1, Beckman), CD4-FITC (clone RPA-T4, BD Biosciences), CD8-PE (clone RPA-T8, BD Biosciences), and NKp46 (clone BAB281, Beckman). The cells were incubated for 30 min at 4°C in the dark, washed twice with 2% FBS in phosphate-buffered saline and analyzed by flow cytometry. After gating on CD3 lymphocytes, the CD3+ population was separated into CD4+ and CD8+ T cells. Natural killer cells were gated out from the CD3- population. All data were acquired using a FACS Canto II instrument (BD Biosciences) and analyzed with FlowJo software version 9.4.10 (Tree Star Inc) and DIVA software version 6.2 (BD).

### Ethics Statement

All studies were approved by the local ethics committee “Comité de Protection des Personnes Ouest IV-Nantes” and the “Agence française de sécurité sanitaire des produits de santé”. Written consent was obtained from all patients and healthy donors.

## Supporting Information

Video S1 **Representative movie from microscopy time series showing PBMCs from a healthy donor infected with GFP-tagged C. albicans cells (MOI of 200∶1).** The cells were illuminated at day 0 and every 10 min over 72 h of incubation with a 300 W xenon lamp fitted with a 488 nm excitation filter. Emission at 515 nm was used for analysis of C. albicans fluorescence with a Leica DMI6000B camera cool Snap HQ2 and processed with Metamorph imaging software version 7.7.4.0.
(AVI)

Video S2 **Movie from confocal microscopy series showing a granuloma six days after infection with GFP-tagged C. albicans (MOI of 2000∶1).** Actin filaments were stained with rhodamine-conjugated phalloidin and nuclear DNA was stained with Hoechst. Fluorescence-stained sections were examined under a Nikon A1 RSI microscope with 20× magnification at constant Z-steps of 1 µm. Laser confocal system comprises a 65 mW Multi-Ar laser (405, 488 and 551-nm excitations are possible). 3D Images were processed with NIS elements version 3.21 (Nikon Instruments Inc.) and Volocity 3D Image Analysis Software version 6.01 (PerkinElmer). Image size: 1212 µm.
(AVI)

Video S3 **Movie from confocal microscopy series showing large green-fluorescent hyphae of C. albicans granulomas. Image size: 1212 µm.**
(AVI)

Video S4 **Representative movie from confocal microscopy showing mature macrophages (Mf) interacting with C. albicans hyphae.** Mature macrophages were characterized by their increased cytoplasmic size and by their ruffled cell membranes that link the adjacent cells and C. albicans. Image size: 100 µm.
(AVI)

Video S5 **Representative movie from confocal microscopy showing mature macrophages (Mf) interacting with C. albicans hyphae. Image size: 125 µm.**
(AVI)

Video S6 **Representative movie from confocal microscopy showing a granuloma six days after co-culture of human PBMC and PMN with GFP-tagged C. albicans at a MOI of 2000∶1.** Actin filaments were stained with rhodamine-conjugated phalloidin and nuclear DNA was stained with Hoechst. 3D Images were processed with Volocity 3D Image Analysis Software version 6.01 (PerkinElmer). Image size 2112 µm.
(AVI)
